# Development of quality indicators and data assessment strategies for the prevention of central venous catheter-related bloodstream infections (CRBSI)

**DOI:** 10.1186/s12879-015-1200-9

**Published:** 2015-10-21

**Authors:** Anke Bramesfeld, Stephanie Wrede, Klaus Richter, Mareike Steen, Björn Broge, Jürgen Pauletzki, Joachim Szecsenyi

**Affiliations:** AQUA Institute for applied quality improvement and research in health care GmbH, Maschmühlenweg 8-10, 37073 Göttingen, Germany; Institute for Epidemiology, Social Medicine and Health System Research, Hannover Medical School, Carl-Neuberg-Straße 1, 30625 Hannover, Germany; Department of General Practice and Health Services Research, Universitätsklinikum Heidelberg, Voßstraße 2, 69115 Heidelberg, Germany

**Keywords:** Infectious disease, Quality of care, Quality assurance, Central venous line, Sepsis

## Abstract

**Background:**

The number of catheter related bloodstream infections (CRBSI) could be reduced and the outcome improved if specific standards in the quality of care were maintained. Therefore, the development of quality assurance (QA) procedures was commissioned to be included in the national mandatory QA programme in Germany.

**Methods:**

Indicators representing quality deficiencies and potential for improvement of quality in relation to prevention and management of central venous catheters (CVC) were developed by 1) evidence-based literature searches and the compiling of an indicator register; 2) a multi-professional expert panel including patient representatives who selected indicators from this register by using a modified RAND/UCLA Appropriateness Method; 3) defining methods for data assessment, risk adjustment and feedback of indicator results to service providers; and 4) consulting all relevant medical societies and other stakeholders with regard to the QA procedures that had been developed.

**Results:**

Thirty-two indicators for CRBSI prevention and management were eventually approved by the expert panel. These indicators represent quality of care at predefined points with regard to indication, insertion and care of CVCs, management of sepsis, general hygiene and training of health care personnel. Fourteen indicators represent processes, together with 7 representing structures and 11 outcomes. For assessing these indicators, data was obtained from four sources: claims data from health insurance funds, routine claims data from hospital electronic information systems, case specific longitudinal documentation from service providers and cross-sectional annual assessment of structures.

**Conclusions:**

It was possible to develop indicators for mandatory QA procedures on CRBSI that take into account the different perspectives of all stakeholders involved. Despite efforts to use routine data for documentation wherever possible, most indicators required extra documentation.

**Electronic supplementary material:**

The online version of this article (doi:10.1186/s12879-015-1200-9) contains supplementary material, which is available to authorized users.

## Background

Inserting a central venous line or catheter (CVC) is often vital for saving the lives of critically ill patients. However, the procedure always carries the risk of secondary infection [1] that, in the majority of cases, is caused by pathogens of the skin. Although only a small number of nosocomial infections are associated with CVCs, they are considered to be a problem as far as patient safety is concerned since CVC associated infections are related to high mortality [2, 3]. According to studies conducted in the U.S. [4–6], it is estimated that up to two thirds of catheter-related bloodstream infections (CRBSI) could be prevented by implementing a number of appropriate preventive measures in line with evidence-based recommendations for indication, placement and care of CVCs [7]. However, with regard to hospitals in Germany, surveys have revealed that evidence-based measures to prevent CRBSI are insufficiently implemented in German hospitals [8, 9].

To address this situation, the Federal Joint Committee (*Gemeinsamer Bundesausschuss*, FJC), the highest joint decision-making body of the joint self-government of physicians, dentists, hospitals and health insurance funds in Germany, decided on the development of national quality assurance (QA) procedures for CRBSI in October 2011. These procedures will be part of the national mandatory QA programme that assesses and benchmarks the quality of all hospitals in Germany.

Below, we will describe the process of identifying indicators that should be evidence-based as well as being tailored to suit the specific requirements of the health system concerned and which should be implemented on a mandatory basis. We are seeking to describe the process in this way so that it can serve as a reference for other working groups that are aiming to identify indicators for measuring the quality of care in relation to the prevention and management of CRBSI and which could be applied to a QA programme at health system level.

## Methods

### Administrative framework

In 2009, the FJC agreed on an initiative for comprehensive improvement of quality assurance across health care sectors in Germany (*Sektorenübergreifende Qualitätssicherung im Gesundheitswesen*, cross-sectoral QA) and commissioned an independent institution (the Institute for Applied Quality Improvement and Research in Health Care (AQUA Institute)) with its development and implementation [10]. The tasks of the AQUA Institute within the cross-sectoral QA programme included the development of national QA procedures, the implementation of data collection processes, the analysis of national data and the feedback of results from data analyses to health care providers to stimulate quality improvement [11]. As cross-sectoral QA is mandatory by law, as set forth in the German Social Code (*Sozialgesetzbuch*), Book V, all health care providers concerned are required to record and transfer data. Quality is measured by indicators that relate to specific clinical areas such as transplantation, neonatology or community-acquired pneumonia.

### Development process

**Fig. 1:**
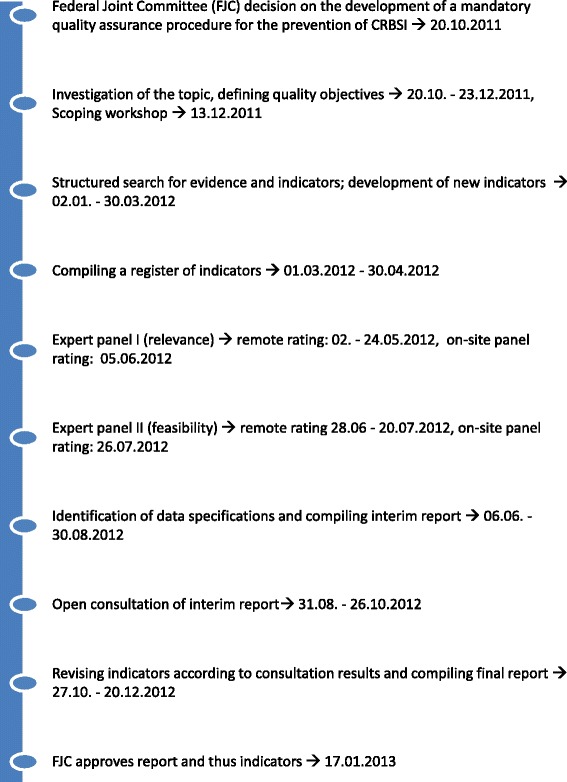
Timeline of the process of developing indicators for quality assurance (QA) procedures for the prevention and management of central venous catheter-related bloodstream infections (CRBSI) that should be mandatory

The development of new QA procedures for, e.g., CRBSI, follow a strict methodology that is approved by the FJC and is applied to the development of all indicators within cross-sectoral QA [12]. Indicators are identified orienting at the “RAND/UCLA Appropriateness Method” [13]. The RAND/UCLA method was originally designed to identify appropriate medical and surgical interventions. Our task, however, was to identify indicators that would measure the presence of appropriate structures, processes and outcomes. We, therefore, took the RAND/UCLA methodological framework in respect of compiling evidence and conducting panel ratings and filled it with our own contents that were tailored to the requirements of the task. The complete procedure from the development of indicators to piloting is summarized in Table 1 and by a timeline given in Fig. 1. Included in the development of indicators are the following steps:

1. Investigation of topic including defining quality objectives and holding a scoping workshop;
2. Structured search for evidence, feasible data sources for indicators and existing indicators, and the development of new indicators;
3. Compiling a register of indicators;
4. Expert panel ratings and agreement on a set of indicators;
5. Identification of data specifications; and
6. Consultation of proposed indicators.

**Table 1:** Phases and tasks in the development and testing of indicators for central catheter-related bloodstream infections (CRBSI)

Phases	Tasks
1. Compiling evidence	● Investigate the topic
	- Exploring care pathways
	- Defining quality objectives
	- Consulting with experts (Scoping workshop)
	● Structured search:
	- Structured search for indicators
	- Structured search for aggregated evidence
	- Systematic literature search
2. Compiling a register of indicators	● Defining each indicator:
	- Numerator, denominator
	- Inclusion and exclusion criteria
	- Target levels or standards if available
	- Type (process, outcome, intermediate outcome, structure)
	- Data sources
	- Evidence
	- Risk adjustment if applicable
3. Panel rating	● Inviting and selecting experts
	● Preliminary meeting
	- Overview of the rating procedure
	- Providing indicator templates
	● Rating rounds
	- Round 1: Relevance and comprehensibility – remote and on-site rating
	- Round 2: Feasibility – remote and on-site rating
4. Identification of data specification	● Specifying each indicator, data sources and required data fields
	● Defining trigger criteria to identify patients for the QA procedure
	● Defining data fields required for risk adjustment
5. Consultation	● Summarizing agreed indicators, data assessment methodologies and implementation plan in an interim report
	● Sending this report to all relevant medical societies and the self-governing associations of physicians, hospitals and health insurance funds for open consultation
	● Revising indicators, data assessment methodology and implementation plan if applicable
	● Compiling final report, that is approved by the Federal Joint Committee (FJC)

#### Investigation of topic and scoping workshop

At this stage, health service pathways are explored that are related to the topic - in this case to CRBSI. The aim is to identify quality deficiencies in health service provision and also potential areas for improvement. Quality objectives are defined and a scoping workshop is organized. The scoping workshop aims at involving stakeholders at an early stage in the QA development process. Thus, the scoping workshop serves as a source for cross-validating the identified service pathways, the possibilities and potential identified for quality improvement. Thus, the groundwork is set for searching and developing indicators following the scoping workshop. The scoping workshop on CRBSI took place in December 2011. Members of a number of medical societies and interest groups involved in providing care related to CRBSI received an invitation either by email or by way of an announcement on our website. Around 90 experts accepted the invitation. Among them were experts from the fields of surgery, internal medicine, intensive care, microbiology, laboratory medicine, infectious disease control and nursing. In addition, representatives from patient organizations, the National Association of Statutory Health Insurance Companies, the German Hospital Federation, the National Association of Statutory Health Insurance Physicians and other stakeholders within the German health care system participated in the workshop.

#### Structured search for indicators

**Fig. 2:**
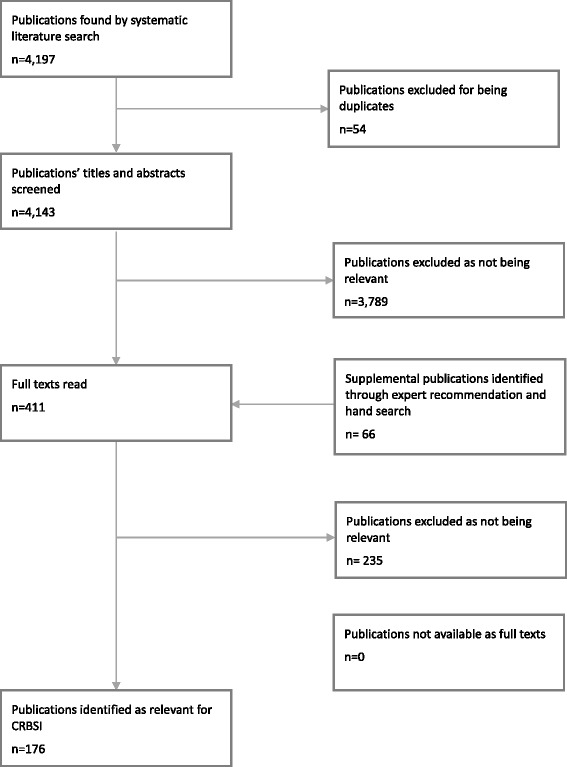
Flow diagram of the systematic literature search for articles on quality indicators for central venous catheter-related bloodstream infections (CRBSI)

Indicators relevant for CRBSI were identified through three searches:

1. A systematic search for indicators related to the quality of care in CRBSI in 72 international indicator databases (January to February 2011). The databases searched are attached to this publication as Additional file 1. Twenty-five relevant indicators were thereby identified.
2. A search for aggregated evidence on CRBSI in the databases of 43 Health Technology Assessment (HTA) agencies. These HTA databases were identified through the International Network of Agencies for Health Technology Assessment (INAHTA). In addition, the database of the Guideline International Network (G-I-N) and two German guideline databases (Association of the Scientific Medical Societies in Germany, AWMF and Agency for Quality in Medicine, ÄZQ) were searched for CRBSI relevant guidelines. The preliminary search revealed 7 HTAs, 22 systematic reviews and 67 relevant guidelines including recommendations by the German Commission for Hospital Hygiene and Prevention of Infection (KRINKO).
3. A systematic search of the literature in Embase using more than 100 predefined search items (20 February 2001, search strategy attached as Additional file 2). The articles were reviewed by two independent researchers. Of the 4143 publications left after removing duplicates, 345 were considered relevant after screening headlines and abstracts. In addition, 66 relevant publications were identified by hand-searching. Thus, 411 publications were eventually reviewed as full-text papers and 175 finally identified as relevant for indicators for CRBSI (see Fig. 2).

Based on the evidence extracted from searches 2 and 3, 65 new indicators were developed.

#### Compiling a register for indicators

The 25 indicators identified in international databases and the 65 that were newly defined were compiled in a register. They were classified according to structure, process or quality outcome and according to the quality dimensions of effectiveness, patient orientation, patient safety, access to and coordination of health care. All indicators concerning patient orientation on access to and coordination of health care had to be newly developed. These quality dimensions had not yet been considered by previous QA procedures.

#### Expert panel rating and agreement on a set of indicators

The indicators compiled then needed to be rated by an expert panel. To identify suitable experts for the panel all medical societies involved in the prevention, diagnosis and treatment of CRBSI were requested to advise their members to apply to become part of the expert panel. In addition, the request was announced at the scoping workshop and published on the cross-sectoral QA website. Forty-eight experts responded to the request of which 13 were selected according to predefined criteria which encompassed the clinical, public health and methodological expertise of the applicant and their association with the relevant disciplines within the care pathway. The panel was finally selected, gathering expertise from internal medicine, anaesthesiology, hygiene and environmental medicine, nursing, haematology, neonatology, infectious disease, microbiology and laboratory medicine. In addition, federal patient organizations delegated two representatives to the panel, making it a group of 15. The participation of patient representatives in all processes concerned with mandatory quality assurance is required by law to ensure that the patient perspective is considered. Patient representatives act on the panel just like any other expert. In the event that the patient representatives lacked specialist knowledge, they were advised by the AQUA Institute.

The panel rated the indicators in two rounds. In the first round, the panel rated the indicators for content validity in terms of *relevance. Relevance* considers an indicator’s impact on clinical outcomes, patient interests and the health system in general. Also considered were the indicator’s ability to distinguish between good and bad quality, and the possibility of actually improving the quality of care that is assessed by the indicators. Subsequently to the first round, the AQUA Institute defined possible data sources and data fields to assess the indicators that the panel had rated positively. In the second round, these indicators were rated according to their *feasibility. Feasibility* considers whether the indicators can, in fact, be implemented from the data sources available, whether the results could be interpreted in respect of quality of care and whether they might cause perverse incentives. The panel, therefore, considered information on data sources and fields, and possibilities for risk adjustment.

During each round, the panellists rated the indicators, first of all remotely and then re-rated the results in an on-site panel meeting. If necessary, quality indicators were modified during the panel meeting before being re-rated.

Similar to the *RAND/UCLA Appropriateness Method* [13], panellists rated the indicators according to different characteristics, on a scale of 1 (the worst) to 9 (the best). All votes counted equally. For each quality indicator, the total panel median scores and the level of agreement within the panel were calculated. Indicators rated with a median score of 7–9 and a consensus of more than 75 % were classified as relevant. Indicators rated with a median score of 1–3 and a consensus of more than 75 % were classified as not relevant. For the feasibility ratings, quality indicators with a median score of more than 4 were classified as feasible.

#### Identification of data specification

For each indicator, data sources and required data fields were specified. This included defining trigger criteria to identify patients for the QA procedures, data fields required for establishing the indicators and also for risk adjustment. Trigger criteria were derived both from the International Classification of Diseases (ICD-10 GM) for outpatient and inpatient sector diagnoses and from the German Operating and Procedure Codes 8 (OPS), a derivate of the International Classification of Procedures in Medicine (ICPM). Furthermore, data fields were defined that could not be derived from routine data and therefore, would need extra documentation by service providers. Finally, specifications for data flow and analyses were provided.

#### Consultation

Indicators, data specifications and data flow models were sent to the FJC and to the medical societies for open consultation. After considering the results of the consultation they were made public in a final report [14].

### Confidentiality and ethics

All participants on the expert panel had to disclose their conflicts of interest before the panel process started. In addition, they signed a statement of confidentiality. The AQUA Institute did not disclose the names of the panel participants until the QA procedures had been finally approved. These measures were taken to minimize the possibility of manipulation of panel participants during the panel process. Ethical approval for the development process, namely the panel but also the scoping workshop, was not required according to the statue of the ethical review committee of the Lower Saxony Medical Association [15]: Developing a QA-procedure including a scoping workshop is not considered research and neither person related data nor patients as such participated. The patient perspective was, however, represented on the panel by delegates from patient organizations.

## Results

The QA procedures to be developed was one that focussed on the handling of conventional CVCs, tunnelled catheters and central venous port systems.

### Final set of quality indicators

Identifying and rating indicators for CRBSI resulted in a final set of 32 quality indicators (Tables 2 and 3). They represent relevant factors along the entire pathway for the prevention and control of CRBSI. Twelve indicators represent relevant specific CVC processes. This starts with the clinical decision for inserting a CVC, the method by which it is inserted, the decision to keep the CVC in place, and the measures that are taken in suspected or proven cases of sepsis. Two further process indicators represent general hand hygiene processes. Eleven indicators represent outcomes, namely sepsis associated with CVCs and finally, the quality of structures related to the prevention of CRBSI is addressed by seven indicators.

**Table 2:** Quality indicators for central venous catheter-related bloodstream infections (CRBSI): indicators to be implemented

No	Indicator	Time-mode for assessment						Data source			Trigger^a^
		At initial treatment (t0)		At follow-up treatment (t1)		Cross- sectional, once a year		Service provider	Hospital claims data	Health insurance claims data	
		Inpatient	Outpatient	Inpatient	Outpatient	Inpatient	Outpatient				
1	Indication for applying CVC	X						X			1
2	Indication for retaining CVC	X						X			1
3	Indication for retaining CVC after transfer to non-ICU-setting	X						X			1
6	Vena femoralis as location for insertion	X						X			1
7	Aseptic conditions upon placement of CVC	X						X			1
8	Criteria for blood culture are met	X						X	X		1
9	Blood cultures taken in the presence of sepsis (as coded by ICD-10) and CVC	X						X	X		1
10	Prevalence of blood cultures in a hospital					X		X			3
16	Explanation or revision of a CVC because of infection			X	X					X	2
19	CRBSI rate in neonatology^b^	X						X			1
20	CRBSI- rate in premature neonates^b^	X						X			1
21	CRBSI rate	X						X		X	1
22	CRBSI rate of multi resistant pathogens	X						X		X	1
23	Use of hand disinfectants in ICU setting					X		X			3
24	Use of hand disinfectants in non-ICU setting					X		X			3
25	Working procedures for applying CVC					X		X			3
26	Working procedures for changing CVC dressing					X	X	X			3
27	Working procedures for applying venous port and connecting it					X	X	X			3
28	Working procedures for handling and applying intravenous fluids					X	X	X			3
29	Working procedures for managing infection in patient with venous port or tunnelled catheter					X	X	X			3
30	Service internal standards for initial treatment with antibiotics					X		X			3
31	Training concept for staff					X	X	X			3
32	Staff participation in training in hygiene and prevention of infections					X	X	X			3

*CVC* central venous line, *CRBSI* central venous catheter-related bloodstream infections

^a^Trigger:

1: Routine claims data from the hospital electronic information systems

2: Claims data from the health insurance companies for which data flow has to be clarified

3: Needs to be defined: annual cross-sectional survey

^b^Will be implemented within QA procedures for premature neonates and neonatology

**Table 3:** Quality indicators for central venous catheter-related bloodstream infections (CRBSI): indicators not recommended for implementation

No	Indicator
4	Indication for applying a tunnelled catheter
5	Indication for applying a venous port
11	Removing CVC after taking blood cultures
12	CRBSI rate – conventional CVC
13	CRBSI rate – tunnelled catheter
14	CRBSI rate – venous port
15	CRBSI rate (according to ICD-10-GM) – venous port and tunnelled CVC
17	CRBSI rate in hemato-oncology units
18	CRBSI rate in non-ICU settings

*CVC* central venous line, *CRBSI* central venous catheter-related bloodstream infections

### Process indicators on indication, insertion, management of sepsis and general hygiene

- Indication: Weighing up the risk of infection and the benefits of a central venous line in the care of critically ill patients is the first relevant step in the chain of procedures related to the prevention of CRBSI. Five process indicators deal with the indications for a CVC: either a conventional CVC (indicator 1), a tunnelled catheter (indicator 4) or a venous port (indicator 5). Indicators 2 and 3 measure the proportion of CVCs that remain in place for more than two days.
- Insertion: Contamination during insertion of a venous line is one of the leading causes of CRBSI [16, 17]. The risk is highest if the vena femoralis is chosen as location for the insertion and, therefore, should be avoided as much as possible. Indicator 6 measures the use of this location; all other locations for insertion were not considered by indicators. Maintaining aseptic conditions during venous puncture is crucial [6, 18, 19] and is, therefore, represented by indicator 7.
- General hygiene: The majority of nosocomial infections are transmitted by contaminated hands. Hand disinfection is non-specific, but highly effective measure to control the risk of nosocomial infection in CRBSI [20, 21]. Hand hygiene was operationalized by the amount of hand disinfectants used in Intensive Care Unit (ICU) and non-ICU settings (indicators 23 and 24).
- Participation in Training: Evidence strongly suggests that training health care personnel in hygiene measures helps to decrease the number of infections [20–22]. Thus, indicator 32 represents participation in training on hygiene and the prevention of infection.
- Sepsis: In cases of suspected sepsis, there are several procedures relevant to risk management. These include taking blood cultures (indicators 8–10), the removal of CVCs (indicator 16), and after having taken blood cultures (indicator 11).

### Outcome indicators on the rate of CRBSI

The relevant outcome to be measured – the actual CRBSI rate – is distinguished according to

- catheter system (indicators 12–15);
- medical specialties with focus on specific risk settings such as haemato-oncology (indicator 17) or non-ICU settings (indicator 18);
- neonates (indicators 19, 20); and
- sepsis in general and sepsis caused by multi-resistant pathogens (indicators 21, 22).

### Structure indicators on work procedures and training

- Work procedures: Whether or not the availability of work procedures and standards should be measured, was highly debated since the mere availability of procedures and standards does not guarantee that they will automatically be used. The panel agreed on indicators that report the availability of work procedures for inserting CVCs, for changing CVC dressings, for puncturing and connecting venous ports, for preparing intravenous-fluids and for taking measures in the event of infection (indicators 25–29). In addition, it was agreed that specific service standards should be available for the initial treatment with antibiotics (indicator 30).
- Concept for training: As guidelines recommend that CVCs should only be handled by trained personnel [22–24], the panel agreed on an indicator depicting the availability of a concept for training in indication, insertion and care of CVCs (indicator 31).

### Excluded indicators

Out of the 90 indicators that were compiled in the registry, the panel excluded 58 indicators as not being relevant. The reason for this was their concern that an indicator could create perverse incentives, such as removing a CVC after a defined time despite the absence of any signs of infection and, unclear evidence, such as the superiority of the vena subclavia over the vena cephalica as the preferred place for CVC insertion. Other indicators were rejected because they would measure interventions that are not available in the majority of places such as antibiotic stewardship services.

All indicators on patient information were rejected. It was felt that those that deal with patient information on hygiene would be better included in a patient survey that is planned for a later stage.

### Indicators not recommended for implementation

Even if indicators were rated as relevant and feasible they were not necessarily recommended for implementation (see Table 3). After the panel process was finalized, the AQUA Institute reviewed all indicators again for their feasibility for implementation in mandatory quality assurance, as conducted under current legislation. In addition, the complete development process underwent consultation with all the relevant medical associations and other stakeholders in the field. As a result, 9 out of the agreed 32 indicators were finally not recommended for implementation, mostly for reasons of documentation efficiency.

### Implementing and assessing data

As Table 2 shows, data for all but one indicator will be assessed by extra documentation from service providers. However, this extra documentation will be enhanced by data from routine claims data from the hospital electronic documentation systems. For example, the indicators on the outcome “sepsis” (indicators 21 and 22) and the indicators on taking blood cultures (indicators 8 and 9) will be documented by service providers as the denominator. The numerator (all patients with a CVC), however, will be drawn from routine claims data from the hospitals. Indicator 16 regarding explanation or revision of a CVC due to sepsis is the only indicator that can be assessed by claims data from the health insurance funds.

All indicators that refer to neonates (19, 20) will be assessed within the already implemented QA procedures on neonatology and premature neonates [25].

Data for indicators 1, 2, 3, 6 and 7 that deal with the indication for CVC and with the process of inserting it, will be solely collected by extra documentation from the service providers. After the consultation process with stakeholders it was decided that these indicators will be collected only for a sample of patients to limit the documentation burden.

All indicators on structures and some process indicators, including those on the use of hand disinfection (indicators 25 and 26), are to be assessed across sectors once a year by in- and outpatient services.

### Feedback

The already well established feedback mechanism in cross-sectoral QA will be used for the above-mentioned QA procedures. This includes an individual annual benchmark report to each service provider that treats patients with CVC. This report will outline the performance of the service provider in respect of defined reference ranges and in comparison with the mean of all other service providers. Service providers that are outside the reference ranges will need to justify their data.

Furthermore, parts of the results will be published annually in an overall federal report, in a report that presents regional results, and in mandatory hospital reports [26, 27].

### Final report

The final report comprised the detailed description of the methodology, the final set of quality indicators and data specification for each indicator. It was approved by the FJC in January 2013.

## Discussion

National QA procedures for CRBSI were developed that focus on potential quality and deficiencies in the service pathway for the prevention and management of CRBSI. Therefore, 32 indicators that measure structure, process and outcome were developed. In addition, we identified data specifications for the final set of indicators, including methods of data collection, data analysis, risk adjustment as well as options for the feedback of assessment results to health care service providers. The way these QA procedures for CRBSI were developed differed in some key aspects from the methodology that had been used until then:

- The indicators concentrate on potential quality and deficiencies only instead of trying to depict the complete service chain as in prior QA procedure developments [28]. This resulted in a smaller but more concise and focused set of indicators.
- Possible data sources for indicators were identified early in the process, before considering eligibility of the indicators. Thus, the panel only discussed indicators that in theory could also be implemented within the context of mandatory QA in Germany. By keeping the set of indicators small and focused, and by clarifying data sources early on, this helped contribute to developing QA procedures that could, in fact, be implemented.
- Unlike other clinical areas in German mandatory QA that address a defined group of services providers (such as transplant surgeons or nurses), the procedures for CRBSI address a variety of clinical specialists. Therefore, it is more difficult than with conventional QA procedures to assign accountability for quality outcomes to a specific service provider.

Below, we will discuss other key aspects of the QA procedure development process.

### Rating indicators by an expert panel

Indicators for CRBSI in the German health care system were selected orienting at the “RAND/UCLA Appropriateness Method” [13]. This method systematically combines scientific evidence and expert opinion, thus taking into consideration clinical and health system realities. Like other authors before, we experienced that the multidisciplinary composition of the panel stimulated interaction in the consensus meetings which resulted in a more comprehensive set of indicators [29]. Discussing the indicators within this panel lead, on the one hand, to the experts changing and modifying their opinions which finally made it possible to reach agreement on a common set of indicators despite initial disagreement. On the other hand, with the panel methodology, there is always the risk of group dynamics or dominant individuals who may influence the results.

Candidate indicators, together with underlying evidence, were presented to the panellists. It is remarkable how indicators that were supported by high-level evidence-based guideline recommendations, were generally agreed upon unanimously by panel members.

It has been debated as to whether participants of indicator-rating panels, who are usually expert clinicians, are qualified to rate the feasibility of indicators and to address operational issues of indicator implementation, such as the time and effort of data processing that is required after data has been collected [30]. Assessment of feasibility might, in part, be beyond the scope of clinical experts as these are generally not experts on data collection and analysis [31]. However, in the process of rating feasibility, the experts made a lot of suggestions towards modifying the indicators which subsequently helped to further improve them and make them more focused. The experts’ rating of the feasibility of indicators is only the first step. The selection of indicators provided by the experts needs to be confirmed by data collection specialists and this then needs to be tested in practice using a validated testing protocol [6, 20].

Within cross-sectoral QA, special emphasis is placed on the patient’s perspective. Therefore, two patient representatives were part of the expert panel. This is noteworthy, as patient participation in defining indicators seems to be rather uncommon [32]. However, the patient’s perspective on quality in health care and on how it should be assessed may differ between patients [33]. Therefore, by including only two patient representatives, this might not have been sufficient to provide a comprehensive reflection of patients’ views. Similar problems were observed in other cross-sectoral QA procedures. Therefore, for future QA procedure development, the possibility of establishing separate focus groups with patients should be considered to supplement cross-sectoral QA methods [34].

### Indicator data specification

Identifying indicators is not the end of the process of developing QA procedures. Without first specifying measurement methodologies and algorithms, and addressing questions of data flow, data protection and data processing, QA procedures cannot be put into practice. In particular, this is the case when it is necessary to compare the combination of data sources from different health care providers with variable data availability. We had planned originally to use more claims data from the health insurance funds. However, this data was often rather unspecific. For example, claims data reporting the insertion of a CVC and the presence of sepsis, does not necessarily mean that sepsis was caused by the CVC. Therefore, claims data needs to be supplemented by extra documentation, e.g. for the outcome indicator *CRBSI Rate* (Indicator 21), the denominator (presence of a CVC) will be taken from routine claims data from the hospitals, while the numerator (sepsis in relation to CVC) will be additionally documented. However, our experience shows that additional documentation usually reveals lower prevalence rates than is found in routine data. Taking the numerator from additional documentation will probably underestimate the number of cases of CRBSI.

To what extent the envisaged data assessment methods will really work needs to be tested in practice (see Table 1). Practice testing of indicators prior to implementation should evaluate indicators for the relevant characteristic including validity, reliability, feasibility and sensibility to change [35]. Only 10–20 % of quality indicators developed for different clinical conditions is reported to have been scrutinized in practice tests [31]. Despite the availability of protocols for indicator practice testing [36, 37], technical specifications of measurement are sparse [38]. Practice testing also includes considering confounding factors due to case mix in hospitals and socio-demographic variables [31]. This risk adjustment is important for a reliable interpretation of indicator results and to prevent risk avoidance on the part of health care providers [39].

### Strengths and limitations of QA procedures

The main strength of these newly-developed QA procedures is the fact that they have been developed orienting at the *RAND/UCLA Appropriateness Method* and that they have included the expert opinions of many different perspectives, including those of patients. By including a number of different experts and by consulting the stakeholders later on in the process, this increases the possibility that the procedures will work effectively and be accepted when implemented in clinical reality. Furthermore, nationwide politically supported cross-sectoral QA has the potential to provide valid quality data on CRBSI for a complete health system. Additionally, the data assessment methods developed in this study which include the use of routine data can provide case-mix adjusted quality information protecting health care providers from an inequitable appraisal of their performance.

Limitations of these newly-developed QA procedures relate to the need for mainstreaming data entry from all hospitals. Furthermore, for each step of implementing QA procedures, consent with the FJC needs to be sought which is – like most policy processes – time consuming.

## Conclusion

QA procedures for CRBSI have been developed that are based on indicators and are meant to be implemented throughout Germany within national mandatory guidelines of QA. This national QA programme aims at holding service providers accountable for the quality of their care that they provide and at improving quality and transparency of care.

This publication is meant to be of use also for others seeking to improve the quality of care in their health systems by implementing indicator-based quality assurance. During the process of indicator development and, particularly, with regard to the identification of relevant indicators, we involved all stakeholders including patient representatives. By doing this and also by consulting the medical associations at a later stage this increases the possibility that adopted QA procedures will really work and be accepted when implemented into clinical life.

Although the need for extra documentation was kept to a minimum as far as possible, meaningful QA procedures on CRBSI cannot solely be based on routine data from hospitals and health insurance companies. There will always be the need for a certain amount of additional documentation. Measures were taken to keep the need for documentation to a minimum by concentrating on the most relevant indicators and by assessing only a sample of patients. Practice testing reveals the feasibility as well as the validity and reliability of newly-developed QA procedures. This practice testing has already been commissioned by the FJC. Results are expected in 2015.

## Additional files

Additional file 1:
**International Indicator databases searched for indicators related to the quality of care for prevention and management of central venous catheter-related bloodstream infections (CRBSI).** (DOCX 29 kb) Additional file 2:
**Search strategy for Embase literature search on the evidence of the quality of care in the prevention and management of central venous catheter-related bloodstream infections (CRBSI).** (DOCX 27 kb)

## Abbreviations

AQUA Institute: Institute for Applied Quality Improvement and Research in Health Care
CRBSI: Central venous catheter-related bloodstream infections
QA: Quality assurance
CVC: Central venous catheter
RAND/UCLA Appropriateness Method: Proper name of a methodology developed by the RAND Cooperation in collaboration with the University of California at Los Angeles to synthesize scientific literature and expert opinion on health care topics
FJC: Federal Joint Committee, Gemeinsamer Bundesausschuss
HTA: Health Technology Assessment
G-I-N: Guideline International Network
KRINKO: Kommission für Krankenhaushygiene und Infektionsprävention [Commission for Hospital Hygiene and Prevention of Infection]
ICU: Intensive Care Unit
