# Supplementary material for: Development of quality indicators and data assessment strategies for the prevention of central venous catheter-related bloodstream infections (CRBSI)
Source: BMC Infect Dis. 2015 Oct 21;15:435. doi: 10.1186/s12879-015-1200-9 (PMC4618155; doi:10.1186/s12879-015-1200-9)
Supplement: Additional file 1: — International Indicator databases searched for indicators related to the quality of care for prevention and management of central venous catheter-related bloodstream infections (CRBSI). (DOCX 29 kb) [file 12879_2015_1200_MOESM1_ESM.docx]

**Additional material file 1:** International Indicator databases searched for indicators related to the quality of care for prevention and management of central venous catheter-related bloodstream infections (CRBSI)

| Indicators database | Indicators were identified |
| --- | --- |
| AAMC – American Association of Medical Colleges (USA) |  |
| Accreditation Canada (Canada) | x |
| ACHS – Australian Council on Healthcare Standards (Australia) | x |
| ÄZQ – Ärztliches Zentrum für Qualität in der Medizin (Germany) |  |
| AHRQ – Agency for Healthcare Research and Quality (USA) |  |
| AIHW – Australian Institute of Health and Welfare (Germany) |  |
| ANA – American Nurses Association´s Safety and Quality Initiative (USA) |  |
| ANAES – Agence nationale d’accréditation et d’évaluation en santé (France) | x |
| ANQ – Nationaler Verein Qualitäts­entwicklung in Spitälern und Kliniken (Switzerland) |  |
| AOK – AOK-Indikatoren für Arztnetze (Germany) |  |
| AQUA – AQUA-Institut (Germany) | x |
| AQUIK – Ambulante Qualitätsindikatoren und Kennzahlen (Germany) |  |
| ACSQHC – Australian Commission on Safety and Quality in Healthcare (Australia) |  |
| AGHA – Australian Government of Health and Ageing (Australia) |  |
| BQS – Institut für Qualität und Patientensicherheit (Germany) |  |
| CAHPS – Consumer Assessment of Healthcare Providers and Systems (USA) |  |
| CIHI – Canadian Institute for Health Information (Canada) |  |
| Care Quality Commission (Great Britain) |  |
| CCECQA – Comité de Coordination de l’Evaluation Clinique et de la Qualité en Aquitaine (France) |  |
| CDC – Centers for Disease Control and Prevention (USA) | x |
| CIAP – Clinical Information Access Programme (Australia) |  |
| CMS – Center for Medicare and Medicaid Services (USA) |  |
| Commonwealth Fund (USA) |  |
| Department of Health (Great Britain) |  |
| DNIP – Det Nationale Indikatorproject (Denmark) |  |
| Dr. Foster Intelligence (Great Britain) |  |
| ECHI – European Community Health Indicators |  |
| EPA – Europäisches Praxisassessment – Qualitätsmanagement in der Arztpraxis (Germany) | x |
| ESQH – European Society for Quality in Healthcare (European) |  |
| FACCT – Foundation of Accountability (USA) |  |
| FOQUAL – Forum de la Qualité (Switzerland) |  |
| GeQiK – Geschäftsstelle Qualitätssicherung im Krankenhaus bei der Baden-Württembergischen Krankenhausgesellschaft (Germany) | x |
| G-IQI – Helios Qualitätsindikatoren (Germany) | x |
| HCC – Health Care Choices (USA) |  |
| Health Canada (Canada) | x |
| Health Council of Canada (Canada) |  |
| HGRD – Health Grades Inc. (USA) |  |
| HHS – US-Department of Health and Human Services (USA) |  |
| IHI – Institute for Healthcare Improvement (USA) |  |
| IPSE – Improving Patient Safety in Europe (Europe) | x |
| IQIP – International Quality Indicator Project (international) | x |
| JCAHO – Joint Commission on Accreditation of Health Care Organizations Hospital Core Measures (USA) |  |
| Kaiser Permanente (USA) |  |
| Leapfrog – Leapfrog Group for Patient Safety (USA) |  |
| Maryland Hospital Association – Quality Indicator Project (Great Britain) |  |
| Massachusetts Health Care Quality and Cost Council (USA) |  |
| National Board of Health and Welfare – Socialstyrelsen (Sweden) |  |
| NCQA – National Committee for Quality Assurance (Great Britain) |  |
| NHS – The Information Centre (Great Britain) | x |
| NHS – Clinical and Health Outcomes Knowledge Base (Great Britain) |  |
| NHS – National Institute for Health and Clinical Excellence: Guidelines (Great Britain) |  |
| NHS North West (Great Britain) |  |
| NHS Quality and Outcomes Framework – QOF (Great Britain) |  |
| NICS – National Institute of Clinical Studies (Australia) |  |
| NQMC – National Quality Measures Clearinghouse (USA) |  |
| NSW Health – New South Wales Government (Australia) | x |
| NVHRI – National Voluntary Hospital Reporting Initiative (USA) |  |
| NRZ – Nationales Referenzzentrum für Surveillance von nosokomialen Infektionen (Germany) | x |
| OECD – Organisation of Economic Cooperation and Development (international) |  |
| OSHPD – Office of Statewide Healthplanning and Development (USA) |  |
| Ontario Ministry of Health and long-term Care (Australia) | x |
| Public Health Agency of Canada (Canada) |  |
| QISA – Qualitätsindikatorensystem für die ambulante Versorgung (Germany) |  |
| QUINTH – Der Qualitätsindikatoren-Thesaurus des GKV-Spitzenverbandes (Great Britain) |  |
| RAND – Research and Development (USA) |  |
| Rhode Island Health Quality Performance Measurement and Reporting Program (USA) | x |
| SARI – Surveillance der Antibiotika-Anwendung und der bakteriellen Resistenzen auf Intensivstationen (Germany) | x |
| Swedish National Institute of Public Health – Statens folkhälsoinstitut (Sweden) |  |
| Verein Outcome (Switzerlanc) |  |
| VGHI – Victorian Government Health Information – Clinical indicators in Victoria’s hospitals (Australia) | x |
| WIDo – Wissenschaftliches Institut der AOK – Qualitätssicherung mit Routinedaten (Germany) |  |
| Zentrum für Qualität in der Pflege (Germany) |  |
