# Supplementary material for: Development of quality indicators and data assessment strategies for the prevention of central venous catheter-related bloodstream infections (CRBSI)
Source: BMC Infect Dis. 2015 Oct 21;15:435. doi: 10.1186/s12879-015-1200-9 (PMC4618155; doi:10.1186/s12879-015-1200-9)
Supplement: Additional file 2: — Search strategy for Embase literature search on the evidence of the quality of care in the prevention and management of central venous catheter-related bloodstream infections (CRBSI). (DOCX 27 kb) [file 12879_2015_1200_MOESM2_ESM.docx]

**Additional material file 2:** Search strategy for Embase literature search on the evidence of the quality of care in the prevention and management of central venous catheter-related bloodstream infections (CRBSI)

| Target population |  | Service/intervention |  | Relevant fields for evaluation |
| --- | --- | --- | --- | --- |
| 'central venous catheter'/exp OR  (central:ab,ti AND venous:ab,ti AND catheter*:ab,ti) OR 'intravenous catheter'/exp OR  'intravenous catheter':ab,ti OR  'intravascular catheter'/exp OR  'subclavian vein catheter'/exp OR  'central line':ab,ti OR 'central venous line':ab,ti OR  (central:ab,ti AND vein:ab,ti AND catheter*:ab,ti) OR 'central venous catheterization'/exp | AND | 'infection prevention'/exp OR  (infection:ab,ti AND prevent*:ab,ti) OR  (sepsis:ab,ti AND prevent*:ab,ti) OR 'infection control'/exp OR  'infection control':ab,ti OR  ( intervention:ab,ti AND infect*:ab,ti) OR ('evidence-based':ab,ti AND intervention:ab,ti) | AND | 'infection'/mj OR infection:ab,ti OR  infections:ab,ti 'bloodstream infection'/exp OR 'bloodstream infection':ab,ti OR  'catheter infection'/exp OR clabsi:ab,ti OR 'bacteremia'/mj OR 'bacteremia':ab,ti OR 'bacteraemia':ab,ti OR 'sepsis'/mj OR  sepsis:ab,ti OR septicemia:ab,ti OR   septicaemia:ab,ti OR 'septicemia'/mj OR 'fungemia'/mj OR 'newborn sepsis'/mj OR 'hospital infection'/exp OR  'bacterial infection'/mj OR  'infectious complication'/mj OR  'infection complication'/exp OR  'device infection'/mj OR  'colonized catheter':ab,ti |
| OR |  | OR |  | OR |
| 'implantable port system'/exp OR  'implantable port':ab,ti OR  ('parenteral nutrition'/exp AND catheter*:ab,ti) OR ('parenteral nutrition':ab,ti AND catheter*:ab,ti) OR ('parenteral nutrition'/exp AND port:ab,ti) OR ('parenteral nutrition':ab,ti AND port:ab,ti) OR 'home intravenous therapy'/exp OR  'home intravenous therapy':ab,ti OR ('chemotherapy'/exp AND port:ab,ti) OR  (chemotherapy:ab,ti AND port:ab,ti) OR ('chemotherapy'/exp AND catheter*:ab,ti) OR  (chemotherapy:ab,ti AND catheter*:ab,ti) OR 'central venous port':ab,ti OR  (port:ab,ti AND catheter*:ab,ti) |  | 'antisepsis'/exp OR 'disinfection'/exp OR 'skin decontamination':ab,ti OR 'hygiene'/mj OR 'hospital hygiene'/exp OR 'hand washing'/exp OR  'antiinfective agent'/mj |  | ('mortality'/mj AND infect*:ab,ti) OR ('survival'/mj AND infect*:ab,ti) OR ('survival rate'/exp AND infect*:ab,ti) OR ('morbidity'/mj AND infect*:ab,ti) OR ('quality of life'/exp AND infect*:ab,ti) OR ('quality of life':ab,ti AND infect*:ab,ti) |
| OR |  | OR |  | OR |
| 'catheter-related':ab,ti |  | (antibio*:ab,ti AND stewardship:ab,ti) OR (antimicro*:ab,ti AND stewardship:ab,ti) |  | 'clinical indicator'/exp OR  'clinical indicator':ab,ti OR  'clinical indicators':ab,ti OR  'quality indicator':ab,ti OR  'quality indicators':ab,ti OR  ('health care survey'/exp AND infect*:ab,ti) OR 'performance measurement system'/exp OR  'health care assessment':ab,ti |
|  |  | OR |  | OR |
|  |  | 'diagnostic procedure'/exp OR  'blood culture'/exp OR  'blood culture':ab,ti OR  'blood cultures':ab,ti OR  'early diagnosis'/exp OR  'clinical observation'/exp OR  'catheter culture':ab,ti OR  'catheter cultures':ab,ti |  | 'patient satisfaction'/exp OR  'patient satisfaction':ab,ti OR  ('patient assessment'/exp AND infect*:ab,ti) OR  ('patient assessment':ab,ti AND infect*:ab,ti) OR  ('patient safety'/exp AND infect*:ab,ti) OR ('patient safety':ab,ti AND infect*:ab,ti) OR 'patient safety indicator':ab,ti OR  'patient safety indicators':ab,t |
|  |  | OR |  | OR |
|  |  | (surveillance:ab,ti AND infection*:ab,ti) OR ('disease surveillance'/exp AND infection*:ab,ti) OR ('observational method'/exp AND infection*:ab,ti) OR  'hospital infection surveillance system':ab,ti OR  'krankenhaus infections surveillance system':ab,ti  OR 'national nosocomial infections surveillance':ab,ti |  | 'methicillin resistant staphylococcus aureus infection'/exp OR  'vancomycin resistant enterococcus'/exp OR  'extended spectrum beta lactamase producing enterobacteriaceae'/exp OR  'multi-resistant':ab,ti OR 'antibiotic resistance'/mj |
|  |  | OR |  |  |
|  |  | 'health care personnel'/mj OR  'hospital personnel'/exp OR  'staff training'/exp OR 'education'/mj OR 'education program'/exp OR 'teaching'/mj |  |  |
|  |  | OR |  |  |
|  |  | 'treatment indication'/exp OR  indication:ab,ti OR  'treatment contraindication'/exp OR contraindication:ab,ti OR  'treatment duration'/exp OR  'dwell time':ab,ti OR 'catheter use':ab,ti OR 'unnecessary use':ab,ti |  |  |
|  |  | OR |  |  |
|  |  | ('vein puncture'/EXP AND infect*:ab,ti) OR 'port placement':ab,ti OR  'insertion technique':ab,ti OR  ('catheter placement':ab,ti AND infect*:ab,ti) OR 'insertion site':ab,ti OR 'catheter type':ab,ti OR 'bandage'/exp OR 'catheter removal'/exp OR  'catheter care'/exp |  |  |
|  |  | OR |  |  |
|  |  | 'hospital discharge'/exp OR  'patient referral'/exp |  |  |
|  |  | OR |  |  |
|  |  | 'eradication therapy'/exp OR  (mrsa:ab,ti AND eradication:ab,ti) OR  (mrsa:ab,ti AND screening:ab,ti) OR decolonization:ab,ti |  |  |
|  |  | OR |  |  |
|  |  | 'quality control'/mj OR  'quality control':ab,ti OR  'quality assurance':ab,ti OR 'total quality management'/exp OR  'quality improvement':ab,ti OR  'medical audit'/exp OR  'clinical competence'/exp OR  'evidence based nursing'/exp OR 'evidence based medicine'/exp OR  'risk management'/exp OR  'case management'/exp OR  'practice guideline'/exp OR  'health care quality'/exp |  |  |
|  |  | OR |  |  |
|  |  | 'patient participation'/exp OR  'patient participation':ab,ti OR  'informed consent'/exp OR  'informed consent':ab,ti OR  'patient decision making'/exp OR 'patient decision making':ab,ti OR 'patient compliance'/exp OR  'patient compliance':ab,ti OR  'patient education'/exp OR  'patient education':ab,ti OR  'patient information'/exp OR  'patient information':ab,ti |  |  |
|  |  | OR |  |  |
|  |  | 'health care delivery'/mj OR  'ambulatory care'/exp OR 'day care'/exp |  |  |
|  |  | OR |  |  |
|  |  | 'infection risk'/exp OR  'infection risk':ab,ti OR ('risk factor'/exp AND infect*:ab,ti) OR ('risk'/exp AND factor:ab,ti AND infect*:ab,ti) OR  ('risk reduction'/exp AND infect*:ab,ti) OR ('risk reduction':ab,ti AND infect*:ab,ti) OR  ('risk assessment'/exp AND infect*:ab,ti) OR  ('risk adjustment:ab,ti AND infect*:ab,ti) |  |  |
|  |  | OR |  |  |
|  |  | 'medical documentation'/exp OR 'medical documentation':ab,ti |  |  |
| Limits: ([article]/lim OR [article in press]/lim)) AND ([english]/lim OR [german]/lim AND [humans]/lim AND [abstracts]/lim) | | | | |
